# Opioid substitution and antagonist therapy trials exclude the common addiction patient: a systematic review and analysis of eligibility criteria

**DOI:** 10.1186/s13063-015-0942-4

**Published:** 2015-10-21

**Authors:** Brittany B. Dennis, Pavel S. Roshanov, Leen Naji, Monica Bawor, James Paul, Carolyn Plater, Guillaume Pare, Andrew Worster, Michael Varenbut, Jeff Daiter, David C. Marsh, Dipika Desai, Zainab Samaan, Lehana Thabane

**Affiliations:** Department of Clinical Epidemiology and Biostatistics, McMaster University, 1280 Main Street West, Hamilton, ON L8S 4L8 Canada; Schulich School of Medicine and Dentistry, University of Western Ontario, 4, 1465 Richmond Street, London, ON N6G 2M1 Canada; Michael G. Degroote School of Medicine, McMaster University, 1280 Main Street West, Hamilton, ON L8S 4L8 Canada; McMaster Integrative Neuroscience Discovery and Study Program, McMaster University, 1280 Main Street West, Hamilton, ON L8S 4L8 Canada; Department of Anesthesia, McMaster University, 1280 Main Street West, Hamilton, ON L8S 4L8 Canada; Canadian Addiction Treatment Centres, 13291 Yonge Street, Richmond Hill, ON L4E 4L6 Canada; Department of Medicine, Hamilton General Hospital, 237 Barton St East, Hamilton, ON L8L 2X2 Canada; Northern Ontario School of Medicine, Ramsey Lake Road, Sudbury, ON P0M Canada; Population Genomics Program, Chanchlani Research Centre, McMaster University, 1280 Main Street West, Hamilton, ON L8S 4K1 Canada; Peter Boris Centre for Addictions Research, St. Joseph’s Healthcare Hamilton, 100 West 5th Street, Hamilton, ON L9C 0E3 Canada; Department of Psychiatry and Behavioural Neurosciences, McMaster University, 1280 Main Street West, Hamilton, ON L8S 4K1 Canada; Centre for Evaluation of Medicine, 25 Main Street West, Hamilton, ON L8P 1H1 Canada; System Linked Research Unit, 175 Longwood Road, South Hamilton, L8P 0A1 Canada

**Keywords:** Opioid addiction, Opioid dependence, Opioid use disorder, Generalizability, Comorbidity, Psychiatric comorbidity, Methodology, Methadone maintenance treatment

## Abstract

**Background:**

Eligibility criteria that result in the exclusion of a substantial number of patients from randomized trials jeopardize the generalizability of treatment effect to much of the clinical population. This is important when evaluating opioid substitution and antagonist therapies (OSATs), especially given the challenges associated with treating the opioid-dependent population. We aimed to identify OSAT trials' eligibility criteria, quantify the percentage of the clinical population excluded by these criteria, and determine how OSAT guidelines incorporate evidence from these trials.

**Methods:**

We performed a systematic review to identify the eligibility criteria used across trials. We searched Medline, EMBASE, PsycINFO, Web of Science, Cochrane Library, Cochrane Clinical Trials Registry (CTR), World Health Organization International CTR Platform Search Portal, and the National Institutes of Health CTR databases from inception to January 1, 2014. To quantify the effect of trials' eligibility criteria on generalizability, we applied these criteria to data from an observational study of opioid-dependent patients (n = 394). We then accessed the Canadian, American, British, and World Health Organization (WHO) OSAT guidelines to evaluate how evidence is used in the recommendations.

**Results:**

Among the 60 trials identified the majority (≥50 % of trials) exclude patients with psychiatric (60 %) and physical comorbidity (51.7 %). Additionally, we found 19 trials exclude patients with current alcohol/substance-use problems (31.7 %), and 29 (48.3 %) exclude patients taking psychotropic medications. These criteria were restrictive and in some cases rendered 70 % of the observational sample ineligible. North American OSAT guidelines made strong recommendations supported by evidence with poor generalizability. National Institute of Health and Care Excellence (NICE) and WHO guidelines for opioid misuse provide a critical assessment of the literature used to inform their recommendations.

**Conclusions:**

Trials assessing OSATs often exclude patients with concurrent disorders. If the excluded patients respond differently to treatment, results from these trials are likely to overestimate the true effectiveness of OSATs. North American guidelines should consider these limitations when drafting clinical recommendations.

**Electronic supplementary material:**

The online version of this article (doi:10.1186/s13063-015-0942-4) contains supplementary material, which is available to authorized users.

## Background

Opioid addiction is a chronic disorder with several risk factors contributing to its development and treatment course [1–3]. The global impact of opioid use is apparent and according to the United Nations Office on Drugs and Crime there were an estimated 26.4 to 36 million (0.03 % global population) people engaging in illicit opioid use in 2012 [4]. Within the US alone 2.1 million people are estimated to be suffering from a prescription opioid addiction [5]. The cost of opioid addiction to both patients and society is high, with estimates in the range of 55.7 billion US dollars [6]. A recent investigation from the RAND provides assessments of the costs of opioid addiction and estimate a range from €2,627 to €60,665 per person, per year. These estimates are comprised of data from American, Australian and Canadian populations and quote the most generalizable estimates encompassing the highest scope of costs (health care, lost worker productivity) at €21,904 per person per year [7].

Despite its high prevalence and global impact, [8] there remain few medication-assisted treatments for opioid addiction. The treatments are known collectively as opioid substitution and antagonist therapies (OSATs) and include both opioid agonist and antagonist treatments. Methadone, buprenorphine, buprenorphine-naloxone and naltrexone, [9] are among the regularly used treatments for addiction patients, with methadone remaining the most commonly prescribed [9]. Patients with opioid dependence are among the hardest to manage and retain in treatment [10, 11]. Their transient lifestyle, extensive social issues, and physical comorbidities contribute to the difficulties of treatment. Less than 15 % of methadone patients successfully finish their treatment as intended, [10, 11] and those who leave treatment have high susceptibility for human immunodeficiency virus (HIV), relapse, and death [10, 11]. When faced with the responsibility of treating such complex and vulnerable populations we are expected to trust the recommendations put forward in the clinical guidelines.

Guidelines rely heavily on the evidence from stringently designed trials and meta-analyses to inform their recommendations [12–15]. Strict eligibility criteria can limit the generalizability of clinical research and leave clinicians guessing as to the effectiveness of interventions in many of their patients [16]. For instance, trials on treatments for opioid dependence commonly exclude patients with psychiatric comorbidities [17–25] in an effort to reduce non-compliance and determine whether a treatment works under optimal conditions. Such selective exclusion of difficult-to-treat patients may inflate the benefits we expect from our treatments. Patients with opioid dependence are known for having comorbid physical and psychiatric disorders [26–31] and the presence of such disorders can adversely affect their outcomes [32–34]. For instance, depressed people receiving opioid substitution therapy (OST) are more likely to consume illicit substances compared to counterparts who do not suffer from depression [32]. Since the prevalence of depression among opioid-dependent patients is high, [31] their exclusion from OSAT trials may bias the results and create inflated estimates of treatment effect. The complexities associated with managing opioid-dependent patients demand the need for thorough clinical guidelines. Accordingly, there is an enormous responsibility to ensure guidelines: (1) rely on the up-to-date evidence with high internal validity; (2) provide information on the populations that may respond differently to treatment and; (3) are transparent about the limitations of the evidence and their ability to provide strong recommendations. Assessment of the generalizability of OSAT trials to real practice and identification of important factors limiting generalizability are key steps in translating the evidence generated by these trials into practice.

While the impact of strict inclusion criteria on the generalizability of trial evidence has been previously evaluated in addiction research, [35–40] these studies are focused on alcohol- and cannabis-dependent populations, where the inclusion criteria and clinical implications may vary greatly from that of opioid-dependent populations. To our knowledge no study has investigated the eligibility criteria of OSAT trials. We set out to: (1) identify OSAT trials’ eligibility criteria and other design characteristics; (2) quantify the proportion of the clinical population excluded by these criteria and; (3) determine how the most recent American, Canadian, British, and World Health Organization (WHO) OSAT treatment guidelines incorporate evidence from these trials.

## Methods

### Review

The protocol for this systematic review has been previously registered (Prospero ID: CRD42013006507) and described elsewhere [41]. The protocol for this review details a larger network meta-analysis currently in progress [41]. Briefly, we performed a systematic review to identify all randomized controlled trials (RCTs) evaluating the effect of a substitute opioid therapy for improving treatment response in opioid-dependent patients. We searched Medline, Excerpta Medica DataBase (EMBASE), PsycINFO, Web of Science, Cochrane Library, Cochrane Clinical Trials Registry (CTR), WHO International CTR Platform Search Portal, and the National Institutes of Health (NIH) CTR databases from inception to January 1, 2014. We asked each primary investigator listed on the NIH Clinical Trial Registry from studies deemed eligible from the title screen of the NIH trial library to submit a list of publications resulting from their trial. We also hand searched all Cochrane reviews evaluating the effectiveness of any OSAT to identify additional studies.

We included only studies evaluating the effectiveness of any opioid agonist or antagonist substitution therapy in patients with opioid addiction. Studies evaluating the effect of OSAT on specialized populations including cocaine- or alcohol-dependent patients were excluded. We will highlight again that the trials used for this review were identified as part of a larger network meta-analysis aiming to evaluate the impact of different OSTs in the general opioid addiction patient populations. Studies included were required to investigate the efficacy of an OSAT using one or more of the outcomes of interest: illicit substance use, treatment attrition, criminal behavior, mortality, physical and psychological well-being as well as adverse events. We placed no age or language restrictions on our search. However, we did require all studies be primary investigations with direct comparison groups (separated by a treatment or placebo). No studies evaluating a single treatment were included (e.g., cohort, cross-sectional studies). We extracted information on the stated trial objectives, eligibility criteria, and study design. All trials eligible for inclusion were subject to risk of bias assessment using the Cochrane Risk of Bias Tool [42]. We used the kappa statistic to assess inter-rater agreement, [43], which is preferable to percent agreement calculations since it takes into account any agreement occurring by chance. Kappa values range from 0 to 1, with values closer to 1 indicating a higher level of agreement [43]. Disagreements during the screening and abstraction process were resolved by consensus. This review adheres to the reporting standards set out by the Preferred Reporting Items for Systematic reviews and Meta-Analyses (PRISMA) guidelines [44].

We then applied trials’ reported eligibility criteria to a sample of 394 opioid-dependent patients participating in the GENetics of Opioid Addiction (GENOA) study, a collaboration between McMaster University and the Canadian Addiction Treatment Centres (CATC).

### GENetics of Opioid Addiction (GENOA) prospective cohort study design

GENOA is an ongoing observational study of the genetic, biological, and social determinants of treatment response for opioid-dependent patients. The GENOA population is made up of opioid addiction patients actively receiving methadone treatment at a CATC facility. The CATC (formerly known as the Ontario Addiction Treatment Centre) are the largest network of methadone addiction treatment facilities in North America – with over 12,000 patients and 57 clinical sites across Canada. Over the past 20 years CATC has treated over 50,000 patients.

Although changes were made to enhance the study’s feasibility and internal validity, the findings and details of methodology employed during the GENOA pilot phase are described elsewhere [45]. Changes made following the pilot phase include: relaxing eligibility criteria, utilizing a prospective cohort design with a follow-up duration of 12 months, and integrating the use of validated tools such as the Brief Pain Inventory (BPI) to assess pain [46], the Mini International Neuropsychiatric Interview version 6.0 (MINI) [47] to assess for psychiatric comorbidities, and the Maudsley Addiction Profile (MAP) instrument to assess addiction severity across personal, physical, and social functioning domains [48]. To be eligible for GENOA participants were required to be at least 18 years old and able to provide informed consent.

GENOA participants are opioid addiction patients receiving methadone for the treatment of opioid dependence. GENOA participants are recruited directly from CATC methadone treatment facilities. Participants provide an initial blood sample as well as addiction severity, pain, and psychiatric assessment at the baseline interview. Participants also provide urine samples to assess for substance use. Over the course of their 12-month follow-up GENOA participants are asked to partake in additional addiction severity assessments using the MAP [48] as well as urine toxicology screening at 3-month intervals.

The CATC is made of up 57 clinical facilitates administering pharmacological therapies for opioid addiction including methadone and buprenorphine. The CATC program offers clinical services including clinical interview to assess opioid dependence according to Diagnostic and Statistical Manual of Mental Disorders, Fourth Edition (DSM IV) criteria, medical examination and laboratory tests, where appropriate, supervised urine testing and initiation of treatment plan. CATC comprises the largest network of addiction treatment facilitates in North America treating over one-third of Ontario’s opioid addiction population. From rural northern Ontario to populous downtown Toronto there is substantial variability in the geographic and economic populations CATC serves, offering a healthy case mix of patients being recruited into the GENOA study. The social, economic, and geographical discordance between CATC sites (both in general and those included in GENOA) increases our confidence in the generalizability of this sample.

We searched www.guidelines.gov for the most recent American and Canadian guidelines with the terms “opioid dependence,” “opioid addiction,” and “opioid substitution treatment.” These search terms were used individually for three separate searches. We also used the National Institute for Health and Care Excellence (NICE) to identify the most recently published clinical guidelines for substitute opioid therapies used by the National Health Service in the UK. WHO guidelines were accessed directly from http://whqlibdoc.who.int/publications/2009/9789241547543_eng.pdf. We extracted information on recommendations made by each guideline, the grade assigned to that recommendation, evidence cited by the guideline, any cautions regarding clinical subgroup effects, and whether the guideline discussed the populations in which the interventions were tested.

The guidelines were also subject to additional evaluation using specific domains from the Appraisal of Guidelines for Research and Evaluation II (AGREE) Instrument. The AGREE II instrument is a validated and reliable tool used to assess the quality of clinical guidelines [49, 50]. The instrument comprises 23 items organized into six quality domains: scope and purpose; stakeholder involvement; rigor of development; clarity of presentation; applicability; and editorial independence [49]. We chose to assess the guidelines using the AGREE II rigor of development and applicability domains since our guideline-specific objective was to determine how the guidelines incorporate evidence into the development and dissemination of clinical recommendations. Two independent reviewers assessed the guidelines according to the detailed instructions provided for the AGREE II.

### Data synthesis and statistical analysis

We summarized categorical variables as proportions and percentages and continuous variables as means with standard deviations. All analyses were performed using Stata 13.1 [51].

## Results

### Findings from systematic review of common eligibility criteria used across OSAT trials

**Fig. 1:**
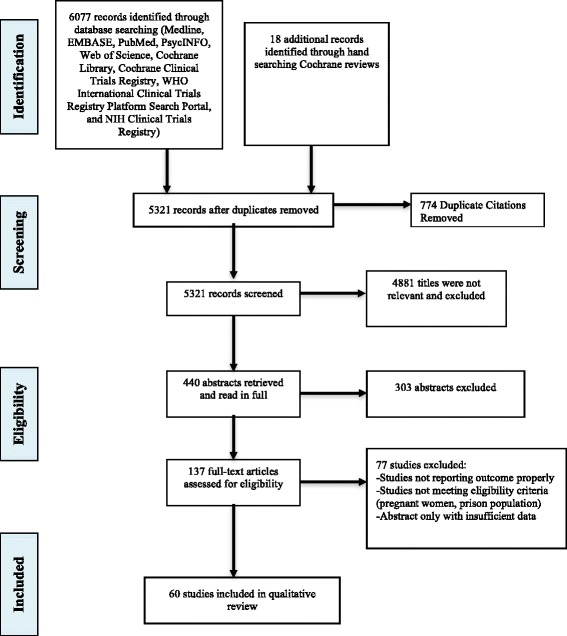
Systematic review study selection flow diagram

Figure 1 outlines the study screening process. We screened 5303 unique articles after removing 774 duplicates with good agreement between reviewers (kappa, 0.71 (standard error [SE] 0.02), 0.85 (SE 0.03), and 0.73 (SE 0.06) for the title, abstract, and full-text screening respectively). The title search was performed in January 2014. A list of the 60 trials included in this review is summarized in Additional file 1. During full-text review 77 articles were excluded, whereby 10 articles identified during the hand-search of Cochrane reviews were duplicates, seven studies did not review an outcome of interest, 36 studies were not randomized trials, one study showed contamination of intervention, six studies used data-linkage/retrospective data design, one study stratified all analyses by sex, and six studies were performed in a specialized population (e.g., cocaine-dependent patients).

Trials identified for inclusion evaluated the effect of methadone, buprenorphine, heroin, naltrexone (oral and implant), combination buprenorphine and naloxone, levo-α-acetylmethadol (LAAM), and morphine. Forty-five percent of trials reported establishing drug efficacy as their primary objective, 20 % reported drug effectiveness as the primary trial aim, and 26 % failed to explicitly state an objective. Table 1 summarizes the eligibility criteria reported across trials, as well as the percentage of opioid-dependent patients from the GENOA sample (n = 394) who satisfy each criterion. Trials often included adult patients (100 %, all 60 trials), meeting the DSM-IV or International Classification of Disease (ICD) criteria for opioid dependence (38.3 %, 23 trials), and exclude patients with psychiatric (60 %, 36 trials) or chronic physical comorbidities (51.7 %, 31 trials). The definitions, measurements, as well as cut-offs used to assess psychiatric and physical functioning varied substantially across trials. While some studies provide a detailed description of conditions that would deem patients ineligible for inclusion, other studies lacked such sufficient detail thus jeopardizing the reproducibility of their trial. For instance, one of the higher quality studies published in the *Lancet* by Schottenfeld et al. (2008) provides a list of conditions they deemed problematic for inclusion into the trial without once discussing how such conditions were measured, “Patients were ineligible if they were dependent on alcohol, benzodiazepines, or sedatives; had concentrations of liver enzymes (alkaline phosphatase or alanine transaminase) greater than three times the upper limit of normal; were dangerous to themselves or others; were psychotic or had major depression; or had life-threatening medical problems,” [19]. This type of description was not uncommon, whereby the majority of studies lacked any explanation of the methods used to assess for different physical conditions or demographic characteristics.

**Table 1:** Summary of eligibility criteria reported across trials

Trial eligibility criteria	Number of trials reporting criteria k (%), (k = 60)	Number of GENOA participants meeting criteria n (%), (n = 394)
Inclusion criteria		
Age ≥18	30 (50.0)	394 (100.0)
DSM-IV or ICD criteria for opioid dependence	23 (38.3)	394 (100.0)
Requirement of previous MMT Treatments or currently receiving methadone	18 (30.0)	394 (100.0)
Provision of positive opioid urine test	18 (30.0)	/
Daily injecting drug use patterns or intravenous drug use behavior	11(18.3)	28 (7.1)
Addiction Severity Score (i.e., >8 on MAP or >12 on ASI)	5 (8.3)	168 (42.6)
Explicit willingness to follow methadone treatment regime	4 (6.7)	394 (100.0)
Educational requirements (high school diploma)	4 (6.7)	352 (89.3)
Abstinence from alcohol and other substances for 1 week prior to study entry	3 (5.0)	/
Reported criminal behavior in last 6 months	1 (1.6)	24 (6.1)
Exclusion criteria		
Presence of psychiatric comorbidity	36 (60)	293 (74.4)
Presence of chronic physical comorbidity	31 (51.7)	225 (57.1)
Presence of acute physical problems	30 (50.0)	106 (26.9)
Presence of psychotropic or neuroleptic medication	29 (48.3)	193 (50.0)
Pregnancy	27 (45.0)	/
Concurrent alcohol or substance abuse problems	19 (31.7)	293 (74.4)
Abnormal liver enzymes	9 (15.0)	/
Presence of HIV	1 (1.7)	3 (0.76)

**/** Indicates this information is not available in the GENOA sample

k indicates trials

Criminal behavior among GENOA participants included: drug trafficking, theft, prostitution, fraud

Psychiatric comorbidity determined using MINI psychiatric evaluation on all GENOA participants (n = 394)

Substance use among GENOA participants included: heroin, benzodiazepine, cannabis, cocaine, crack cocaine, and alcohol

Chronic physical comorbidity was measured in the GENOA sample as a composite outcome of one or more of: HIV, chronic pain, liver disease, hepatitis, diabetes, epilepsy, or other

Acute physical problems determined in GENOA sample using MAP physical symptoms scores (participants reporting <20 were considered to have no acute physical health problems)

*GENOA* Genetics of Opioid Addiction, *DSM-IV* Diagnostic and Statistical Manual of Mental Disorders, Fourth Edition, *ICD* International Classification of Disease, *MMT* methadone maintenance treatment, *MAP* Maudsley Addiction Profile, *ASI* Addiction Severity Index, *HIV* human immunodeficiency virus, *MINI* The Mini International Neuropsychiatric Interview

The most restrictive criteria were the exclusion of patients with psychiatric comorbidities or those using alcohol and other substances, which alone renders 74.4 % of the GENOA participants ineligible for inclusion. While not as commonly reported (<50 % of trials), the application of other restrictive criteria such as the exclusion of patients who take psychotropic medications and the requirement for intravenous drug use behavior would render 50 and 93 % of the sample ineligible for inclusion respectively.

### The use of evidence in clinical practice guidelines

We identified three North American guidelines for the treatment of opioid dependence using the www.guidelines.gov database [12–14]. We identified the UK clinical practice guidelines for opioid maintenance therapy using the NICE database [52]. We also evaluated the WHO 2009 international guidelines for managing opioid-dependent patients [53]. Tables 2 and 3 summarize the recommendations made by each guideline, the grade assigned to that recommendation, evidence cited by the guideline, any cautions regarding clinical subgroup effects, the number of GENOA participants excluded by the combined eligibility criteria reported in the trials cited in the guideline, and whether the guideline discussed the populations in which the interventions were tested.

**Table 2:** Guidelines available for evaluation of the appropriateness of opioid agonist and antagonist treatments for patients with opioid dependence

Title of guideline	Country	Year	Intervention(s) assessed	Outcome	Clinical trial design characteristics in supporting evidence	Use of observational studies	Meta-analyses included individual risk of bias assessment
Clinical practice guideline for management of substance use disorders (SUD) [13]	USA	2009	Methadone and buprenorphine	Decrease in opioid use (relapse prevention)	Explanatory by design with strict eligibility criteria	No	Yes, but no discussion of severe limitations of included trials (allocation concealment)
Clinical practice guideline for management of substance use disorders (SUD) [13]	USA	2009	Methadone and buprenorphine	Retention in treatment	Explanatory by design with strict eligibility criteria	No	No discussion of poor methodological quality of included trials and estimate suffers from imprecision
Clinical practice guideline for management of substance use disorders (SUD) [13]	USA	2009	Naltrexone	Decrease in opioid use (relapse prevention) and retention	Explanatory trials by design with strict eligibility criteria used within meta-analysis	No	Meta-analyses provided (two with proper risk of bias assessment)
Buprenorphine/naloxone treatment for opioid dependence clinical practice guidelines [14]	Canada	2011	Buprenorphine/naloxone	Decrease in opioid use (relapse prevention) and retention	Explanatory trials by design with strict eligibility criteria used within meta-analysis and cited trials	Yes, retrospective chart review and patient registry databases	Yes
Methadone maintenance treatment program standards and clinical guidelines [12]	Canada	2011	Methadone maintenance treatment	Decrease in opioid use (relapse prevention)	Explanatory trials by design with strict eligibility criteria used within meta-analysis and cited trials	Yes	No discussion of methodology of included trials used to inform recommendations. Systematic review meta-analyses are cited.
Methadone and buprenorphine for the management of opioid dependence [52]	UK	2007	Methadone and buprenorphine	Decrease in opioid use (relapse prevention) and retention	Explanatory trials by design with strict eligibility criteria used within meta-analysis and cited trials	Yes, provide evidence from systematic reviews of trials and non-randomized studies	Yes (with discussion of methodological limitations)
Guidelines for the psychosocially assisted pharmacological treatment of opioid dependence [53]	International guidelines	2009	All pharmacological therapies for opioid dependence	Decrease in opioid use, treatment retention	Explanatory trials by design with strict eligibility criteria used within meta-analysis and cited trials	Yes, provide evidence from systematic reviews of trials and non-randomized studies	Yes (with discussion of methodological limitations)

**Table 3:** International guideline assessment of the evidence for substitute opioid therapy in treatment of opioid dependence (assessment of recommendations based on effect reported in literature)

Title of guideline	Evidence provided	Eligibility criteria used across trials (including criteria from trials within meta-analyses used as evidence)	Grading of evidence by guideline panel	Reported net effect of intervention	Recommendation	Guideline provided caution about populations the intervention was assessed in	Cautions	Discussion of opioid substitution treatment use for subpopulations (psychiatric patients, patients on psychotropic medication, patients with concurrent poly-substance use problems)
Clinical practice guideline for management of substance use disorders (SUD) [13] (Methadone and buprenorphine for reduction in illicit opioid use)	[54, 55, 70–77]	Inclusion of patients >18 with DSM-IV diagnosis of opioid dependence Exclusion of patients with psychiatric disorders, concurrent substance use disorders, and those being prescribed psychotropic medications	Good	Substantial^a^	A strong recommendation	No	Note that buprenorphine is preferred to methadone in pregnant women	Yes, methadone was more effective than buprenorphine for patients with concurrent cocaine dependence
Clinical practice guideline for management of substance use disorders (SUD) [13] (Methadone and buprenorphine for patient retention)	[75]	Inclusion of patients >18 with DSM-IV diagnosis of opioid dependence Exclusion of patients with psychiatric disorders, concurrent substance use disorders, and those being prescribed psychotropic medications.	Good	Substantial^a^	A strong recommendation	No	None	No
Clinical practice guideline for management of substance use disorders (SUD) [13] Naltrexone for reduction in illicit opioid use and treatment retention)	[78–80]	Inclusion of patients >18 with DSM-IV diagnosis of opioid dependence Exclusion of patients with psychiatric disorders, concurrent substance use disorders, and those being prescribed psychotropic medications	Poor to fair	Small to moderate	No recommendation for or against the routine provision of the intervention is made. At least fair evidence was found that the intervention can improve health outcomes, but concludes that the balance of benefits and harms is too close to justify a general recommendation.	No	Suggested for use in highly motivated patients	Yes, recommends naltrexone within highly motivated patients
Buprenorphine/naloxone treatment for opioid dependence clinical practice guidelines[14]	[81–84]	Inclusion of patients with daily drug injection behavior, ≥18 and a DSM-IV diagnosis of opioid dependence Exclusion of patients prescribed psychotropic medications, and patients with serious physical conditions or concurrent drug/alcohol dependence	Good	Not reported	A strong recommendation	No	A list of contraindications is provided (e.g., pregnancy, allergy, severe liver dysfunction, acute severe respiratory illness. No mention of psychiatric illness or concurrent substance use problems.	Yes, require no concurrent substance use problems prior to buprenorphine induction as well as required full management of psychiatric symptoms
Methadone maintenance treatment program standards and clinical guidelines [12]	[76, 85–89]	Inclusion of patients ≥18 with DSM-IV diagnosis of opioid dependence 18 and 50 years, history of intravenous opioid dependence, no chronic medical illnesses, absence of a major mental illness, a negative pregnancy test for women, and at least 3 months since the patient’s last treatment at the clinic	Not graded	Not reported	There are guideline suggestions provided but no “rank” of recommendation	No	Note about treatment pregnant women and patients under 18	No
Methadone and buprenorphine for the management of opioid dependence [52]	[82]	Inclusion of patients ≥18 with DSM-IV diagnosis of opioid dependence Exclusion of patients with psychiatric disorders, comorbid substance use, patients on psychotropic medications	Reported as good quality evidence	Not reported	No direct recommendations made	Yes, discussed the populations the interventions were tested in and explicit detailing of trial design characteristics	None	Yes
Guidelines for the psychosocially assisted pharmacological treatment of opioid dependence [53]	[76, 87, 90, 91]	Inclusion of patients age ≥18 meeting DSM-IV criteria for opioid dependence with six prior treatment episodes at the facility running the randomized trial, or a single prior methadone treatment, and urine screen positive for opioids.	Moderate for substance use behavior and high for treatment retention (for both methadone and buprenorphine)	Small to moderate (for both opioid use and retention)	Strong	Yes, also a guidance is provided for managing specific subpopulations (women, patients with psychiatric comorbidity, patients with chronic pain)	Note agonist therapy is suggested most effective, methadone is preferred to buprenorphine. In pregnant women less safety evidence is available, use methadone in such cases.	Yes
		Exclusion of patients with psychiatric or chronic physical comorbidities or being prescribed psychotropic medication, acute medical condition, and pregnant women						

Good evidence refers to high-grade evidence (with at least one properly designed randomized trial) directly linked to health outcome

Poor to fair refers to high-grade evidence (with at least one properly designed randomized trial) linked to intermediate outcome or moderate-grade evidence (evidence obtained from well-designed cohort or case–control analytic studies, evidence obtained from multiple time series studies; dramatic results in uncontrolled experiments) directly linked to health outcome and/or refers to opinions of respected authorities; descriptive studies and case reports; reports of expert committees of evidence or no linkage of evidence to health outcome

*DSM-IV* Diagnostic and Statistical Manual of Mental Disorders

^a^Substantial refers to a more than a small relative impact on a frequent condition with a substantial burden of suffering, or a large impact on an infrequent condition with a significant impact on the individual patient level

The most recent clinical practice guidelines for management of substance use disorders from the US Department of Defense and Department of Veteran Affairs (2009) uses evidence from both trials and observational studies to inform recommendations for methadone and buprenorphine opioid substitution treatment [13]. The recommendations made within the US guidelines were based on evidence from trials excluding patients with psychiatric disorders, [54, 55] concurrent alcohol or poly-substance use, [54–56] and the patients prescribed psychotropic medications [55, 56]. The College of Physicians and Surgeons of Ontario published the most recent methadone guidelines, utilizing a systematic search across five databases and the grey literature [12]. The guideline committee found only four studies to address the issue of methadone effectiveness among prescription opioid users, with large weight placed on a retrospective observational study [57] and a systematic review lacking a risk of bias assessment [58]. Similar to the methadone maintenance guidelines, the Canadian guidelines for buprenorphine rely on evidence from trials and systematic reviews of studies excluding patients on psychotropic medications, and patients with serious physical comorbidities as well as concurrent substance/alcohol dependence [14]. The Canadian guidelines did not provide a description of how they evaluated the individual studies used to support their recommendations [12, 14]. There is no explicit discussion of the risk of bias assessment conducted on the supporting body of evidence [12, 14]. When applying the criteria reported by the trials cited in each guideline we find the Canadian buprenorphine/naloxone guideline [14] are basing their recommendations on the most restrictive evidence, whereby only seven participants from the GENOA sample meet the combined eligibility requirements.

Contrary to the practices used by North American guideline panels, the NICE and WHO clinical practice guidelines for opioid misuse provide an appraisal of the evidence used to inform the guidance and suggest the importance of managing comorbid disorders with the use of psychosocial interventions during maintenance therapy or before using an OSAT to treat opioid dependence [52, 53]. WHO guidelines provide explicit recommendations for managing participants with comorbid disorders (poly-substance use, pregnancy, psychiatric disorders) as well as transparency with respect to the evidence used to inform recommendations [53]. NICE guidelines provide disclaimers for the knowledge users about the populations the interventions were tested in, and the overall limitations for using the evidence to guide recommendations in specific subpopulations [52]. WHO guidelines go so far as suggesting comorbid disorders be addressed by the same consultant, a method used for increasing uptake and adherence to OSAT [53]. While WHO guidelines provided a section detailing recommendations for important subpopulations, they did not discuss any of the important issues of generalizability [53]. Moreover, WHO guidelines went so far as suggesting there was no uncertainty as to the directness of the evidence in their evidence profile for the questions, “Is methadone effective for the treatment of opioid dependence?” despite the concerning stringent inclusion criteria applied across trials [53]. In fact, the only criticisms WHO raised with respect to the generalizability of the evidence were in the case of studies performed on in-patient populations [53].

Table 4 summarizes the application of the AGREE II instrument to the guidelines. A domain-specific score is provided for each guideline in addition to the individual item scores. The UK and WHO guidelines ranked highest for the rigor of development and applicability domains (Table 4). Within this domain, UK and WHO guidelines scored a 93, and 95 respectively. Additionally each guideline (UK and WHO) consistently ranked 6 and 7 (strongly agree). This indicates the European guidelines use systematic methods to identify the supporting evidence in addition to being transparent about guideline development and the limitations of the evidence used to support their recommendations. The North American guidelines ranked considerably lower at 50, 44, and 27 respectively for the United States, Canadian buprenorphine, and Canadian methadone guidelines. WHO and UK guidelines again ranked highest within the applicability domain (scores 93, 86 respectively). The Canadian buprenorphine guidelines did not follow far behind with a domain score of 67. The Canadian buprenorphine guidelines spent considerable time describing the need to move buprenorphine on the all provincial drug benefit plans to ensure access for all opioid-dependent patients [14]. The American and Canadian methadone guidelines fell further behind with domain scores of 25 and 29 respectively. Table 4 provides a summary of the individual scores, allowing us to evaluate where guidelines performed weakest according to AGREE II.

**Table 4:** Application of the rigor of development and applicability AGREE II domains to international guidelines for substitute opioid therapy in treatment of opioid dependence

AGREE II items	Canadian methadone maintenance guideline [12] score	Canadian buprenorphine guideline [14] score	American substance abuse guideline [13] score	NICE substance abuse guideline [52] score	World Health Organization guideline [53] score
Domain III: rigor of development					
Systematic methods were used to search for evidence	2	3	5	7	7
The criteria for selecting the evidence are clearly described	2	2	4	7	6
The strengths and limitations of the body of evidence are clearly described	1	2	2	6	7
The methods for formulating the recommendations are clearly described	3	2	4	6	7
The health benefits, side effects, and risks have been considered in formulating the recommendations	5	5	6	7	6
There is an explicit link between the recommendations and the supporting evidence	4	3	4	6	7
The guideline has been externally reviewed by experts prior to its publication	2	7	1	7	7
A procedure for updating the guideline is provided	2	5	6	6	6
Domain score	27	44	50	93	95
Domain V: applicability					
The guideline describes facilitators and barriers to its application	4	4	2	6	7
The guideline provides advice and/or tools on how the recommendations can be put into practice	3	6	3	6	6
The potential resource implications of applying the recommendations have been considered	2	6	3	7	7
The guideline presents monitoring and/or auditing criteria	2	4	2	5	6
Domain score	29	67	25	86	93

*AGREE* Appraisal of Guidelines for Research and Evaluation, *NICE* National Institute for Health and Care Excellence

## Discussion

This study provides an overview of the limitations of OSAT literature, using multiple resources including results from a well-designed systematic review, [41] and an application of the findings within a clinical sample of opioid-dependent patients [45]. This study also provides a systematic assessment of the guidelines, highlighting the important limitations we can work to improve for the future.

Results from this systematic review suggest trials most often include adult patients meeting the DSM-IV/ICD criteria for opioid dependence with intravenous drug use behavior and a past history of methadone treatment. Trials most often exclude participants having a psychiatric or chronic physical comorbidity, current alcohol or substance use problem, as well as those taking psychotropic medications. When applying these criteria to a clinical sample of methadone patients we found them to be largely restrictive, and in some cases render 70 % of the GENOA sample ineligible. Criteria such as the exclusion of participants with psychiatric or physical comorbidity, concurrent alcohol/substance use problems, as well as those using psychotropic medication appeared to have the largest cost to recruitment, where more than 50 % of the GENOA sample would be lost by the application of such criteria.

The majority of international clinical practice guidelines rely on out-of-date systematic review evidence to inform guidance development as well as making strong recommendations based on many of the trials with strict eligibility that we assessed in our review. Assessment of both Canadian and American guidelines revealed concerning practices, where both panels provide numerous trials as evidence supporting recommendations for different opioid substitution treatments without once discussing the impact of trial eligibility criteria [12, 13]. The guidelines neither acknowledge the restrictive design of the trials or the generalizability of the evidence [12–14]. The guidelines go so far as to rank the quality of the evidence as good, despite the concerning limitations we have raised for each of the cited studies [12, 13]. These issues are highlighted further when we applied the combined eligibility criteria reported by trials cited in the guidelines to the GENOA sample, whereby the highest number of GENOA participants these studies could have been generalized to would include 20 people out of 394.

Additionally, when applying the AGREE II rigor of development and applicability domains we found the North American guidelines performed considerably worse in using systematic search methods to identify research, and reporting the limitations as well as generalizability of the evidence. These practices were contrary to the transparency of reporting found in the WHO and UK guidelines. Use of the Grading of Recommendations Assessment, Development and Evaluation (GRADE) of guidelines criteria is likely impacting the stark quality differences between North American and European guidelines [59]. European guidelines provide transparent appraisal of the evidence used to inform recommendations and even go so far as cautioning the application of the evidence in psychiatric or criminal populations [52]. These findings suggest the need for North American guideline committees to evaluate and impose the critical evidence synthesis approaches utilized by the WHO and NICE.

### Understanding the evidence and the need for change

The use of restrictive eligibility criteria is often set by investigators to ensure the safety for recruited patients to the new intervention being tested and to maximize the chances of observing a treatment effect under optimal conditions. Prior to phase III trials, interventions have never been tested in randomized comparison design. Entry into phase III efficacy trials is governed by restrictive eligibility criteria and conduct is often controlled by rigid protocols. This may explain why many of the trials identified for this review adhered to an explanatory design.

Testing the effect of interventions on highly specific groups may be associated with unintended harm to patients once the intervention is released for use in the general population. Our results indicate the majority of opioid substitution therapy trials exclude participants with major psychiatric disorders. This exclusion criterion is in no way novel, in fact many trials exclude patients with psychiatric comorbidities. What is concerning is the lack of understanding over what may happen to these populations once the drug is released for wider use. For example, varenicline was tested in a randomized double-blind placebo controlled trial to assess its efficacy for reducing smoking [60]. This trial excluded participants with a history of psychiatric comorbidity including: major depression requiring treatment within the past year, panic disorder, psychosis, bipolar disorder, or anorexia nervosa or bulimia [60]. Upon Food and Drug Administration (FDA) approval for use in the general population, many patients began to present with psychiatric symptoms including erratic behavior and suicide attempt [61]. Many now criticize the pharmaceutical company for marketing the use of this drug in the general population before knowing the real effect of the intervention in participants with psychiatric comorbidities, especially since smoking is prevalent among patients with psychiatric disorders. These side effects would have been better noticed had the proper implementation trials taken place, or had the intervention been tested in a more representative sample.

### Future directions

There are important reasons why patients with certain comorbidities (physical, psychiatric) are excluded from trials. Efficacy trials seek to determine whether the intervention actually works under the appropriate conditions. When the objective of a trial is to determine the effect and safety of an intervention an appropriate design would be to test the intervention under optimal conditions. Patients with psychiatric comorbidities, especially those with addictive disorders are known to have difficulty complying with interventions, [62] and the inclusion of these participants can dilute the treatment effects we observe in trials. This trend suggests the results from stringently designed trials reporting small treatment effects are unlikely to withstand the inclusion of non-compliant patients. For instance, results from one trial show a significant hazard ratio (HR) for the comparison of buprenorphine to naltrexone for improvement of patient retention (HR: 1.56; 95 % confidence interval [CI]: 1.01, 2.41) [19]. This trial’s findings are fragile and unlikely to sustain a small number of changes to the reported events across treatment arms, which would be the likely outcome had the trial been performed in a more pragmatic patient population [19].

It is important we address the need for inclusion of non-compliant participants in trials. Recognizing that patients already have challenges complying with the strict methadone treatment regimes [62], this issue is further complicated for trials recruiting addiction patients because of the high prevalence of concurrent psychiatric comorbidities [45], which are known to impact intervention adherence [62]. There have been suggestions for improving compliance among patients with psychiatric disorders; one emphasized in the literature is the use of telephone and electronic reminders [62], which has been demonstrated to improve compliance in non-psychiatric patients [63, 64]. Other ways to improve patient compliance with addiction treatment include focusing on adherence through educational sessions with family health teams, consistent symptom measurement, and appropriate treatment tailoring [65]. Instead of excluding these participants from trials and limiting the generalizability as well as understanding of the treatment within a population representing a good portion of addiction patients, we encourage trialists to design studies with features that help increase patient compliance so to ensure an understanding of interventions effectiveness in the clinical population.

We recognize there are also other important reasons guiding the design of strict protocols, for instance the exclusion of pregnant women or patients with acute physical conditions may be a protective measure in efficacy trials where drug safety is still being determined. However, the real problem arises when we rely exclusively on evidence from these trials before we can determine the effectiveness in a “real world” sample of different types of patients (e.g., patients with comorbidities or on psychotropic medications). The limitations of the strictly designed protocols that govern efficacy and safety of trials have been largely addressed by the introduction of implementation trials, which are investigations whose primary aim is to test treatments using flexible protocols for participant selection and treatment administration. These trials often have lax eligibility criteria with the aim of including the participant we will find in the clinical practice population. However, these trials are not common in the field of addiction medicine, restricting us to the use of stringent efficacy trials to inform clinical guideline development.

Uptake in the planning and commitment to implementation or “effectiveness” research will likely stem from an increase in discussion and acceptance of the need for pragmatic trial designs. The goal in implementation trial design should be to maximize the safety of participants included in the trial while also balancing the applicability of the findings. Pragmatic trials – sometimes called implementation trials – are evaluated on a continuum and should not be characterized by a specific set of criteria [66]. We recommend future trials in addiction medicine need not abandon all “explanatory,” or more stringent designs but instead work to evaluate the intervention in a wide range of participants as a secondary objective. Implementation trials should aim to include those participants with psychiatric comorbidities, poly-substance use disorders, and chronic physical conditions. Provided there are enough patients within each subgroup, researchers may be able to evaluate the mediating impact of each comorbidity and with confidence determine the true impact of physical or psychiatric abnormalities within addiction patients.

#### Limitations

Reliance on a treatment sample of methadone patients may potentially impact the generalizability of this study. Using the GENOA sample of participants provides a unique opportunity to demonstrate the restrictive impact of eligibility criteria reported in the addiction literature. However, demonstrating such an effect requires the use of a generalizable sample of addiction patients. Participants recruited from the CATC comprise a treatment sample, which may in fact have higher levels of comorbidities (both physical and/or psychiatric) than patients earlier on in the cycles of addiction. By the time patients are receiving pharmacological therapy for opioid dependence they are often at a later stage in their addiction course, placing them at higher risk for exposure to HIV, hepatitis, infectious disease, opioid-induced hyperalgesia, and poor social/economic living conditions. In addition, patients may only seek treatment once their physical, psychiatric, or social functioning is seriously impeded. In fact, the GENOA sample may lack the population of patients experiencing the range of problems which often coerce or force individuals into treatment altogether. However, we must also acknowledge that addiction is a complex disorder, often accompanied by serious physical and psychiatric comorbidities. Incident misuse of opioids is known to result from serious physical comorbidities such as pain [67] and from suffering experienced as a consequence of anxiety or depression [68]. Recognizing there may be discordance between prevalent users currently seeking treatment and more “incident” cases, we maintain the clinical profile of incident users also reflects a high degree of mental and physical abnormality. We emphasize the CATC population of patients may be a prevalent sample of addiction treatment-seeking patients, and that the results from these trials are generated to inform the treatment of such populations, and as such it is still important we demonstrate the large effect these criteria may have on weakening the directness of the evidence.

We acknowledge that observational studies are subject to selection bias, whereby patients agreeing to participate in the study may reflect a different population. To evaluate such bias we have elected to compare the demographic and clinical characteristics of participants in the GENOA study to a sample of CATC patients from four economic and geographically diverse clinics. This sample of CATC patients includes population data from four clinics and includes demographic and clinical characteristics data from all patients actively receiving treatment from these sites. Please refer to Table 5.

**Table 5:** Comparison of the demographic and clinical characteristics of GENOA participants to the general population of CATC patients

Demographic and clinical characteristics	CATC population level data, N = 1354	GENOA sample, n = 394	Statistical significance observed testing differences between groups
Mean age (SD)	38.4 (10.7)	38.5 (10.9)	0.87
Sex (percentage male)	66.9	53.3	*p* <0.05
Mean duration on MMT in years (SD)	2.1 (1.2)	4.3 (4)	*p* <0.05
Mean methadone dose in mg per day (SD)	81.9 (53.5)	78.1 (41.2)	0.19
Hepatitis C positive %	29.9	22.3	*p* <0.05
HIV positive %	0.3	0.8	0.19
Marital status (% single, divorced)	64.8	68.2	0.19

Two-sample t test used to assess for differences between groups (CATC and GENOA) for continuous values

Chi-square test used to assess for differences between groups for categorical variables

*GENOA* Genetics of Opioid Addiction, *CATC* Canadian Addiction Treatment Centres, *SD* standard deviation, *MMT* methadone maintenance treatment, *HIV* human immunodeficiency virus

The GENOA sample was largely representative of the general CATC population, whereby the mean age, mean methadone dose (mg/day), prevalence of HIV, as well as marital status was not statistically significantly different between samples. There were, however, some differences, whereby the general CATC patients were shown to have a higher prevalence of hepatitis C and on average a shorter treatment duration. Additionally, the general CATC sample was made up of a higher proportion of men than found in the GENOA population. These results do suggest that the GENOA participants are made up of patients with longer treatment duration and as such these patients are likely susceptible to having a higher number of physical or psychiatric comorbidities. However, these results also suggest the CATC population has a higher level of hepatitis than our sample, which may also suggest the GENOA study may be subject to a “healthy volunteer bias,” whereby sicker patients are less likely to engage in the study. Inadvertently, this would bias our own results toward the null and overall suggests more participants in the “general” active treatment population would be excluded that we are purporting.

In Canada buprenorphine is not covered by the provincial drug insurance plans, and as such these patients reflect either (1) an employed population with benefits covering therapy, or (2) those patients who can afford out-of-pocket coverage. In light of the administrative differences between methadone and buprenorphine coverage, we chose to include only the sample of methadone patients in the GENOA investigation, which could reflect a more marginalized population of drug treatment-seeking patients and is a potential limitation to the generalizability of the GENOA sample.

Information regarding criminal offences, sexual behavior, and domestic conflict were collected by self-report from patients agreeing to participate and thus likely underestimated due to social desirability bias. We acknowledge social desirability bias may impact the estimates for some of the demographic data collected in this study. However, we maintain important variables, such as psychiatric comorbidity, were ascertained using a validated questionnaire, the MINI. In addition, physical comorbidity (diabetes, chronic pain) was evaluated using self-report and confirmed via information logged by attending physicians in the patients’ electronic medical record. Urinalysis was performed to ascertain poly-substance use. We aimed to include as many objective measurements as possible, and when unavailable we relied on safeguards such as electronic medical record confirmation.

Additionally, we may find that the definitions, measurements, and cut-offs (if relying on measurement tools) used to assess for physical and psychiatric functioning across clinical trials may be quite different than those used in the GENOA study. Thus, there is potential that the exclusion criteria reported across trials and later applied to the GENOA sample are being misused. Psychiatric comorbidity can vary from obvious psychotic disorders to any anxiety or depressive disorder. Depending on the thoroughness of the trial investigators and indeed the thoroughness of the clinicians administering the GENOA assessment tools (MINI, BPI, MAP), differing rates of psychiatric problems will be identified and could compromise the aims of our study. Due to the serious limitations in reporting of the definitions and measurements for many of the eligibility criteria discussed across the literature, we caution the interpretation of the application of such criteria using the GENOA study.

Provided we had reliable data on the medical and demographic characteristics of opioid addiction patients, we would be better equipped to demonstrate the clinical guidelines are not appropriate for the US and UK populations. Administrative data provided by Health Maintenance Organizations in the US or the National Health Service in the UK could serve as sources for population-level data. However, the quality of this data is questionable due to the high susceptibility for misclassification. A recent study evaluated the misclassification of psychiatric disorders based on the comparison of medical records and administrative data and found only moderate agreement for any mental comorbidity [69]. We acknowledge the problems associated with opioid dependence are impacted by the health, social, and judicial systems, which can vary across countries. However, to say the prevalence of psychiatric and physical comorbidity, as well as prescription of psychotropic medication varies so much between countries, as well as types of addiction populations such that it would render the larger message of this study insignificant is improbable.

## Conclusions

Findings from our analysis of the literature as well as application of common eligibility criteria to a clinical sample of patients with opioid use disorder demonstrate large differences between the trial and clinical population of opioid-dependent patients. Evaluation of the global context and impact of these findings shows the concerning state of addiction medicine, where we find the majority of studies used to inform clinical practice are not generalizable to the population seen in clinical practice. When more than 50 % of the addiction patient population suffers with comorbid psychiatric conditions, yet only a small fraction of the evidence used to inform the current treatment strategies for these patients is tested on patients with psychiatric conditions we are faced with a critical dilemma. Are we providing the appropriate treatments? Are we completing our due diligence to this patient population? Are we possibly putting our patients at risk? These questions cannot be answered until the appropriate re-evaluation of the evidence takes place using pragmatic trial designs and implementation studies.

## Additional file

Additional file 1:
**Table outlining description of studies included in the systematic review.** (PDF 262 kb)

## Abbreviations

AGREE: Appraisal of Guidelines for Research and Evaluation
ASI: Addiction Severity Index
BPI: Brief Pain Inventory
CATC: Canadian Addiction Treatment Centres
CI: confidence interval
CTR: Clinical Trials Registry
DSM-IV: Diagnostic and Statistical Manual of Mental Disorders, Fourth Edition
EMBASE: Excerpta Medica DataBase
FDA: Food and Drug Administration
GENOA: Genetics of Opioid Addiction
GRADE: Grading of Recommendations Assessment, Development and Evaluation
HIV: human immunodeficiency virus
HR: hazard ratio
ICD: International Classification of Disease
LAAM: levo-α-acetylmethadol
MAP: Maudsley Addiction Profile
MINI: The Mini International Neuropsychiatric Interview
MMT: methadone maintenance treatment
NICE: National Institute for Health and Care Excellence
NIH: National Institutes of Health
OATC: Ontario Addiction Treatment Centres
OSAT: opioid substitution and antagonist therapy
OST: opioid substitution therapy
PRISMA: Preferred Reporting Items for Systematic reviews and Meta-Analyses
RCT: randomized controlled trial
SD: standard deviation
SE: standard error
WHO: World Health Organization
UK: United Kingdom
US: United States
